# Genetic mutations affecting mitochondrial function in cancer drug resistance

**DOI:** 10.1007/s13258-022-01359-1

**Published:** 2023-01-07

**Authors:** Chanhaeng Lee, Sang‑Hee Park, Sungjoo Kim Yoon

**Affiliations:** 1 Department of Biomedicine & Health Sciences, The Catholic University of Korea, 222 Banpo‑daero, Seocho‑ku, Seoul, 065‑591 Republic of Korea; 2 Department of Medical Life Sciences, The Catholic University of Korea, 222 Banpo‑daero, Seocho‑ku, Seoul, 065‑591 Republic of Korea

**Keywords:** Mitochondria targeting drugs, Mutations in mitochondrial proteins, Drug resistance, Chemotherapy

## Abstract

Mitochondria are organelles that serve as a central hub for physiological processes in eukaryotes, including production of ATP, regulation of calcium dependent signaling, generation of ROS, and regulation of apoptosis. Cancer cells undergo metabolic reprogramming in an effort to support their increasing requirements for cell survival, growth, and proliferation, and mitochondria have primary roles in these processes. Because of their central function in survival of cancer cells and drug resistance, mitochondria are an important target in cancer therapy and many drugs targeting mitochondria that target the TCA cycle, apoptosis, metabolic pathway, and generation of ROS have been developed. Continued use of mitochondrial-targeting drugs can lead to resistance due to development of new somatic mutations. Use of drugs is limited due to these mutations, which have been detected in mitochondrial proteins. In this review, we will focus on genetic mutations in mitochondrial target proteins and their function in induction of drug-resistance.

## Introduction

Mitochondria consist of double membranes, the outer mitochondrial membrane (OMM) and the inner mitochondrial membrane (IMM), with intermembrane space in between. Invagination of IMM into the mitochondrial matrix results in formation of cristae, a structure that is essential for the function of mitochondria. Mitochondria serve as a central hub for physiological processes in eukaryotes, including production of adenosine triphosphate (ATP), regulation of calcium dependent signaling, generation of reactive oxygen species (ROS), and regulation of apoptosis (Han et al. 2019).

Mitochondria are dysfunctional in various cancers due to somatic mutations of mitochondrial DNA (mtDNA) and defects of mitochondrial enzyme leading to tumorigenesis and tumor progression due to abnormalities of the metabolic pathway or resistance to apoptosis (Hsu et al. 2016). Generation of ROS, oxidative stress, and other mutations in mtDNA responsible for continued induction of tumorigenesis are exacerbated by mutations of mtDNA (Hahn and Zuryn 2019). Hypoxia-inducible factor 1α (HIF1α), whose overexpression occurs in many cancers as an adaptive regulator of hypoxia for tumorigenesis, is stabilized by defects in metabolic enzymes such as succinate dehydrogenase (SDH) and fumarate hydratase (FH) (Talks et al. 2000; Sharp and Bernaudin 2004; Pollard et al. 2005; Yee Koh et al. 2008). Furthermore, mitochondria have a capacity for rapid sensing and adaptation to stress stimuli, thus management of drug-induced stress can lead to resistance to chemotherapy (Mizutani et al. 2009; Eisner et al. 2018).

Cancer cells undergo metabolic reprogramming in an effort to support their increasing requirements for cell survival, growth, and proliferation, and mitochondria have primary functions in these processes. Because of their central function in survival of cancer cells and drug resistance, mitochondria are an important target in cancer therapy (Ghosh et al. 2020; Vasan et al. 2020). Many drugs target mitochondria. However, development of new somatic mutations, leading to resistance, can occur with continued use of mitochondrial-targeting drugs (Aminuddin et al. 2020). These mutations, which have been detected in mitochondrial proteins, limit the use of drugs (Tanaka et al. 2021; Xu and Ye 2022).

In this review, we will focus on genetic mutations in mitochondrial target proteins and their function in induction of drug-resistance.

### Mitochondrial targets for cancer therapy

Development of cancer therapies targeting dysfunctional mitochondria in cancer has been reported (Horton et al. 2008; Kuznetsov et al. 2011; Thomas et al. 2017; Kim et al. 2020). Binding of chemotherapeutic drugs such as doxorubicin, trastuzumab, and sunitinib to mtDNA leads to induction of apoptosis through generation of ROS and loss of mitochondrial function (Gorini et al. 2018). However, these chemotherapeutic drugs exhibit a high level of toxicity and serious adverse effects on heart function have been reported (Khakoo et al. 2008; Chatterjee et al. 2010; Huszno et al. 2013).

Alternatively, compared to the conventional chemotherapy, utilization of drugs targeting mitochondrial proteins (Table 1) that induce growth of cancer cells can minimize side effects through selective removal of cancer cells (Dong et al. 2008; McGee et al. 2011; Zamberlan et al. 2022). Oligomerization of pro-apoptotic proteins, BAX/BAK is inhibited by members of the anti-apoptotic B cell CLL/lymphoma 2 (BCL-2) family of proteins located in the inner mitochondrial membrane, which block the release of mitochondrial cytochrome c, consequently resulting in inhibition of apoptosis. Overexpression of these proteins, which are associated with chemotherapy resistance, occurs in many cancer cells (Kang and Reynolds 2009). Use of anti-apoptotic BCL-2 family protein inhibitors such as venetoclax, navitoclax, Obatoclax, TW-37, BM-1197, S63845, and AZD-5991 for induction of apoptosis in cancer cells has been reported (Neuzil et al. 2013; Ashkenazi et al. 2017; Kotschy et al. 2016; Tron et al. 2018; Cournoyer et al. 2019; Ahn et al. 2019; Sun et al. 2019). Glycolytic proteins can be targeted using 3-bromopyruvate (3BP), Mito-CP, Mito-Q, and 2-Deoxyglucose (2-DG) to inhibit energy metabolism for induction of apoptosis in cancer cells (Cheng et al. 2012). Mutated isocitrate dehydrogenases (mIDH) is inhibited by AGI-5198, AGI-6780, AG-120, and AG-221, which are detected in a variety of cancers and are regarded as prime targets for chemotherapy through regulation of the tricarboxylic acid (TCA) cycle (Zong et al. 2016; Golub et al. 2019). Induction of ROS and destruction of cancer cells are induced by alpha-tocopheryl succinate (α-TOS), metformin, rotenone, and Mitochondrially targeted vitamin E succinate (MitoVES) through inhibition of the function of proteins of the electron transport chain (ETC) in oxidative phosphorylation (OXPHOS) activity and generation of ATP (Zhang and Fariss 2002; Li et al. 2003; Dong et al. 2008; Kalyanaraman et al. 2018; Fontaine 2018).

**Table 1:** Drugs of targeting mitochondrial proteins

Mitochondria protein-target drug	Target protein	Cell death mechanism	Reference
AZD-5991	BCL-2 family	Apoptosis induction	Tron et al. (2018)
BM-1197			Sun et al. (2019)
Obatoclax (GX15-070)			Cournoyer et al. (2019)
S63945			Kotschy et al. (2016)
TW-37			Ahn et al. (2019)
Navitoclax (ABT-263)			Ashkenazi et al. (2017)
Venetoclax (ABT-199)			
2-deoxyglucose (2-DG)	Hexokinase II	Inhibition of cell metabolism	Zhao et al. (2019)
3-bromopyruvate (3BP)			Lis et al. (2016)
Benitrobenrazide			Zheng et al. (2021)
FV-429			Zhou et al. (2016)
AG-120	Isocitrate dehydrogenase I, II	Inhibition of cell metabolism	Golub et al. (2019)
AG-221			
AGI-5198			
AGI-7680			
Enasidenib (AG-221)			
Ivosidenib (AG-120)			
Metformin	Complex I	ROS accumulation	Fontaine et al. 2018
Rotenone			Li et al. (2003)
Alpha-tocopheryl succinate (α-TOS)	Complex II	ROS accumulation	Dong et al. (2008)
Atpenin A5			Kluckova et al. (2013)
MitoVES			Yan et al. (2015)
Thenoyltrifluoroacetone			Zhang and Fariss (2002)

Despite their strong efficacy against cancer with low side effects, modulation of the efficacy of these drugs can occur in various ways in cancer cells, leading to treatment resistance. Resistance to mitochondrial protein-targeted anticancer therapies can be caused by various genetic factors in various carcinomas (Xu et al. 2018; Çoku et al. 2022). In addition, induction of apoptosis can be avoided due to involvement of the mitochondrial regulatory protein that induces apoptosis in interactions with various proteins rather than a single protein (Lopez and Tait 2015).

### Cases of resistance to mitochondrial-targeted anti-cancer drugs due to genetic factors

#### Drugs targeting anti-apoptotic proteins to induce intrinsic apoptosis

The intrinsic apoptotic pathway is controlled by the BCL-2 family, a family of proteins sharing BCL-2 homology (BH) domains, through control of mitochondrial outer membrane permeabilization (MOMP). Among the members of the BCL-2 family, anti-apoptotic proteins such as BCL-2, BCL-XL, and MCL-1 induce inhibition of apoptosis leading to tumor promotion (Youle and Strasser 2008). These proteins of the BCL-2 family are important in several carcinomas as targets for cancer therapy, including prostate cancer, breast cancer, and blood cancer (Emi et al. 2005; Yoshino et al. 2006; Soderquist et al. 2016).

Of the BH1 through BH4 domains, BH3 is a key domain for induction of anti-apoptosis in proteins of the anti-apoptotic BCL-2 family (Kelekar and Thompson 1998). Direct activation of BAX/BAK resulting in induction of apoptosis occurs by way of BH3-only proteins, such as the BCL-2 interacting apoptosis mediator (BIM) (O'Connor et al. 1998), and the activities of BAX/BAK are inhibited by BIM binding to the BH3 domain of the BCL-2 family proteins, which results in anti-apoptosis (Ewings et al. 2007). In the effort to maintain the activity of BIM, many studies on cancer therapies using “BH3 mimics” that bind to this domain of BCL-2 family proteins have been reported (Souers et al. 2013; Wang et al. 2016; Konopleva et al. 2016; Campos and Pinto 2019; Fleischmann et al. 2022; Calis et al. 2022). Venetoclax (ABT-199), a representative BH3 mimic, is used for chemotherapy of hematologic malignancies including acute myeloid leukemia (AML) that shows high expression of BCL-2 (Souers et al. 2013; Konopleva et al. 2016; Campos et al. 2018; Fleischmann et al. 2022).

Genetic mutations responsible for resistance to venetoclax were recently detected in patients with progressive Chronic Lymphocytic Leukemia (CLL) with G101V, F104C, F104L, and D103Y mutations in the *BCL-2* gene. These mutations, which reduced the binding affinity of venetoclax to the BCL-2 protein due to the presence of a bulkier sidechain within the interior of globular BCL-2 protein, were responsible for resistance to venetoclax (Birkinshaw et al. 2019; Tausch et al. 2019). BCL-2 and BCL-XL were inhibited by the use of navitoclax (ABT-263) in hepatocellular carcinoma (HCC) cells, however, MCL-1 mRNA and protein were stabilized, resulting in a limited effect (Wang et al. 2014). Because venetoclax and navitoclax are selective drugs against BCL-2, activation of other members of the BCL-2 family such as MCL-1 has been reported as the primary cause of resistance (Van Delft et al. 2006; Souers et al. 2013). In an effort to address this limitation, a combination of S63845 (MCL-1 inhibitor) and venetoclax were applied, resulting in induction of apoptosis in venetoclax-resistant AML cells (Hormi et al. 2020).

In the case of *MCL-1* with L267V mutation detected in myeloma patients, the mutation does not interfere with binding of MCL-1 inhibitors such as S63845 and AZD-5991 to MCL-1, rather it prevents displacement of pro-apoptotic proteins by the drug, resulting in disruption of the process of apoptosis (Chen et al. 2018).

In addition, a decrease in BAX, a pro-apoptotic protein, also led to induction of resistance to venetoclax. Interaction of BAX with Voltage Dependent Anion Channel (VDAC) occurs upon induction of apoptosis, leading to an increase of MOMP, resulting in loss of membrane potential and release of cytochrome c (Adachi et al. 2004). Significantly reduced efficacy of venetoclax was reported in BAX-deficient CLL patients with C-terminal *BAX* mutations who received long-term treatment with venetoclax (Blombery et al. 2020), resulting in elimination of external mitochondrial membrane localization of BAX and induction of resistance to venetoclax (Blombery et al. 2022).

#### Drugs targeting glycolysis for inhibition of mitochondrial metabolic pathways

Most cancer cells undergo metabolic reprogramming in an effort to adapt to unfavorable conditions such as hypoxia and a low supply of nutrients up to 60% of ATP is derived from the “Warburg effect” and the remaining ATP from OXPHOS. The Warburg effect, a high level of glycolysis over OXPHOS even in the presence of oxygen in cancer cells, enables acquisition of oxygen-independent metabolism in cells (Alfarouk et al. 2014; Lis et al. 2016).

Intracellular fixation of glucose catalyzed by hexokinase (HK) is the first step of glycolysis. ATP-dependent phosphorylation of glucose is catalyzed by HK for generation of glucose-6-phosphate (G6P) which is also utilized in OXPHOS and the pentose-phosphate pathway (Rosano et al. 1999).

The mitochondrial bound form of hexokinase 2 (HK2) most likely has an anti-apoptotic function. which might be responsible for its overexpression in most cancers. HK2 is bound to the outer membrane protein VDAC in mitochondria and probably gives HK2 an advantage regarding access to ATP generated during OXPHOS (Nakashima et al. 1986; Arora and Pedersen 1988). HK2 improves the rate of glycolysis and is required for initiation and maintenance of tumors. Therefore, development of anti-cancer drugs that target HK2 for inhibition of glycolysis and induction of apoptosis has been reported (Zhou et al. 2016; Zheng et al. 2021).

2-DG, a glucose analogue, competes with glucose and binds to HK2, leading to inhibition of glycolysis (Zhao et al. 2019). No cases of resistance to 2-DG, an HK2 inhibitor, in cancer have been reported. However, one case of 2-DG resistance with a novel mutation in the yeast gene Hkk2 has been reported (Zhao et al. 2019). No interaction was observed between the Hkk2 G238V mutation and either glucose or 2-DG, however, its potential to affect binding and catalysis of hexose through an allosteric mechanism was demonstrated (Hellemann et al. 2022), suggesting a clinical relevance to 2-DG resistance.

#### Drugs targeting the TCA cycle to inhibit mitochondrial production of ATP

The TCA cycle, also known as the citric acid cycle or the Krebs cycle, is a mediator that assists in production of ATP through supply of electrons to the ETC. The TCA cycle, which occurs in mitochondria, is composed of eight chemical reactions for production of electron reservoirs in the form of NADH and FADH2 from acetyl-CoA derived from carbohydrates, proteins, and fats. In turn, NADH and FADH2 are utilized in the OXPHOS pathway for production of chemical energy in the form of ATP. Both storage of electrons and generation of amino acid precursors occur during this cycle.

Although utilization of aerobic glycolysis is known to be a hallmark of cancer cell metabolism, cancer cells also rely on the TCA cycle for production of energy and macro-molecular synthesis (Anderson et al. 2018; Eniafe and Jiang 2021). Catalysis of isocitrate to α-ketoglutarate (α-KG) and reduction of NAD(P) + to NAD(P)H are induced by Isocitrate dehydrogenases 1 and 2 (IDH1 and IDH2), metabolic enzymes in the TCA cycle (Reitman and Yan 2010). In particular, IDH2, located in mitochondria, is involved in regulation of oxidative respiration, and thus plays an important role in tumorigenesis (Nekrutenko et al. 1998; Kalyanaraman et al. 2018; Qiao et al. 2021). Mutation of *IDH1* and *IDH2* has been reported in several carcinomas including AML, and solid tumors, including glioma, chondrosarcoma, and cholangiocarcinoma (Wouters 2021). The conversion of α-KG to 2-hydroxyglutarate (2-HG), an oncometabolite, is catalyzed by mutant IDH1 and IDH2 (mIDH1 and mIDH2), which also mediate most of the carcinogenic potential (Dong and Neuzil 2019). Accumulation of 2-HG is a factor in tumor formation and growth of malignant tumors (Dang et al. 2009). Therefore, high efficacy of anticancer drugs, including AGI-5198, AGI-6780, AG-120, and AG-221 in targeting mIDH1 and mIDH2 has been reported in a wide range of cancer types (Wouters et al. 2021).

Enasidenib (AG-221), a selective inhibitor of mIDH2, displays a potent inhibition of 2-HG production in the context of the *IDH2* R140Q/WT heterodimer or the *IDH2* R140Q homodimer. Suppressed production of 2-HG and induction of cellular differentiation caused by AG 221 in primary human mIDH2–positive AML cells has been demonstrated (Yen et al. 2017). A recent study reported on the secondary mutations in *IDH2*, Q316E and I319M, detected in two AML patients who developed resistance to Enasidenib and tumor relapsed. The Q316E mutation caused a reduction of hydrogen bonding with Enasidenib, and the I319M mutation conveyed steric hindrance to the bulky side chain (Zhuang et al. 2022). Resistance to Ivosidenib (AG-120), an inhibitor of mIDH1, was also reported in the AML patients with the secondary mutation, S280F of *IDH1*. *IDH1* S280F mutation was expected to result in a steric hindrance caused by substituted phenylalanine near the binding site of Ivosidenib and mIDH1 (Oltvai et al. 2021).

#### Drugs targeting the mitochondrial ETC protein to apoptosis via generation of ROS

ROS, a byproduct of normal metabolism of oxygen in cells, are highly reactive oxygen-containing molecules that react readily with other molecules in cells. Production of ROS occurs through recurring biochemical reactions during OXPHOS in the ETC with passage of electrons through a series of proteins in order to finally reach oxygen in mitochondria. Increased levels of ROS detected in cancer cells has long been regarded as tumorigenic through promotion of genomic instability. A moderately increased level of ROS in cancer cells can promote tumorigenesis and progression through activation of signaling pathways responsible for regulation of cellular proliferation, metabolic alterations, and angiogenesis as well as induction of DNA mutation (Sullivan and Chandel 2014; Perillo et al. 2020).

However, a continuous increase in the level of ROS leads to inactivation of BCL-2 and BCL-XL via activation of c-Jun N-terminal Kinase (JNK) and induction of apoptosis by release of cytochrome c by BAX/BAK (Redza-Dutordoir and Averill-Bates 2016). Therefore, wide use of cancer treatment that induces excessive production of ROS has been reported (Perillo et al. 2020). NADH generated through the TCA cycle in the mitochondrial matrix is oxidized by respiratory complex I (Complex I), which also causes reduction of ubiquinone to ubiquinol using two electrons (Sharma et al. 2009). Catalysis of the oxidation of succinate to fumarate and transfer of electrons to ubiquinone are induced by SDH (Complex II) (Bandara et al. 2021). They donate electrons to ETC and ultimately play an important role in generation of ATP (Nolfi-Donegan et al. 2020). Production of ATP is hindered by inhibition of Complex I and Complex II, which also causes excessive production of ROS (Chen et al. 2007), which is a target of cancer treatment (Yoshida et al. 2021; Kluckova et al. 2013).

Complex I is composed of 45 subunits; seven of these, *ND1-6* and *ND4L*, are encoded by mitochondrial DNA (Sharma et al. 2009). Binding of rotenone, an inhibitor of Complex I, to the *ND4* site, results in induction of apoptosis, inhibiting proliferation of cells in various carcinomas including lung cancer, colon cancer, and breast cancer (Heinz et al. 2017; Kampjut and Sazanov 2020). The G11778A mutation in *ND4* has been reported to induce resistance to rotenone in patients with Leber's hereditary optic neuropathy (Degli Esposti et al. 1994; Musiani et al. 2022). Although no clinical cases in cancer patients have been reported, it is expected that cancer patients with the G11778A mutation of *ND4* will have an equal likelihood of developing resistance to rotenone. In addition, resistance to rotenone was reported in hypoxia-tolerant human glioma cells (M010b) harboring the T14634C mutation of *ND6* that showed no change in expression of *ND6* (DeHaan et al. 2004).

Ubiquinone obtains electrons through binding to the ubiquinone binding site (Qp) of complex II. Upon gaining electrons, ubiquinone is reduced to ubiquonol in order to supply electrons to complexes III and IV (Cecchini 2003; Sun et al. 2005). Interaction of Atpenin and MitoVES with Qp in complex II, which inhibited the reduction of ubiquinone and the oxidation of succinate, has been reported (Miyadera et al. 2003; Yan et al. 2015). According to the result, ROS was produced by Complex II, leading to saturation of the succinate concentration (Siebels and Dröse 2013). However, interaction of Complex II with inhibitors such as thenoyltrifluoroacetone (TTFA), Atpenin A5, and MitoVES was inhibited by mutations at the Qp-binding site of Complex II, leading to development of drug resistance (Kluckova et al. 2015).

## Conclusion

**Fig. 1:**
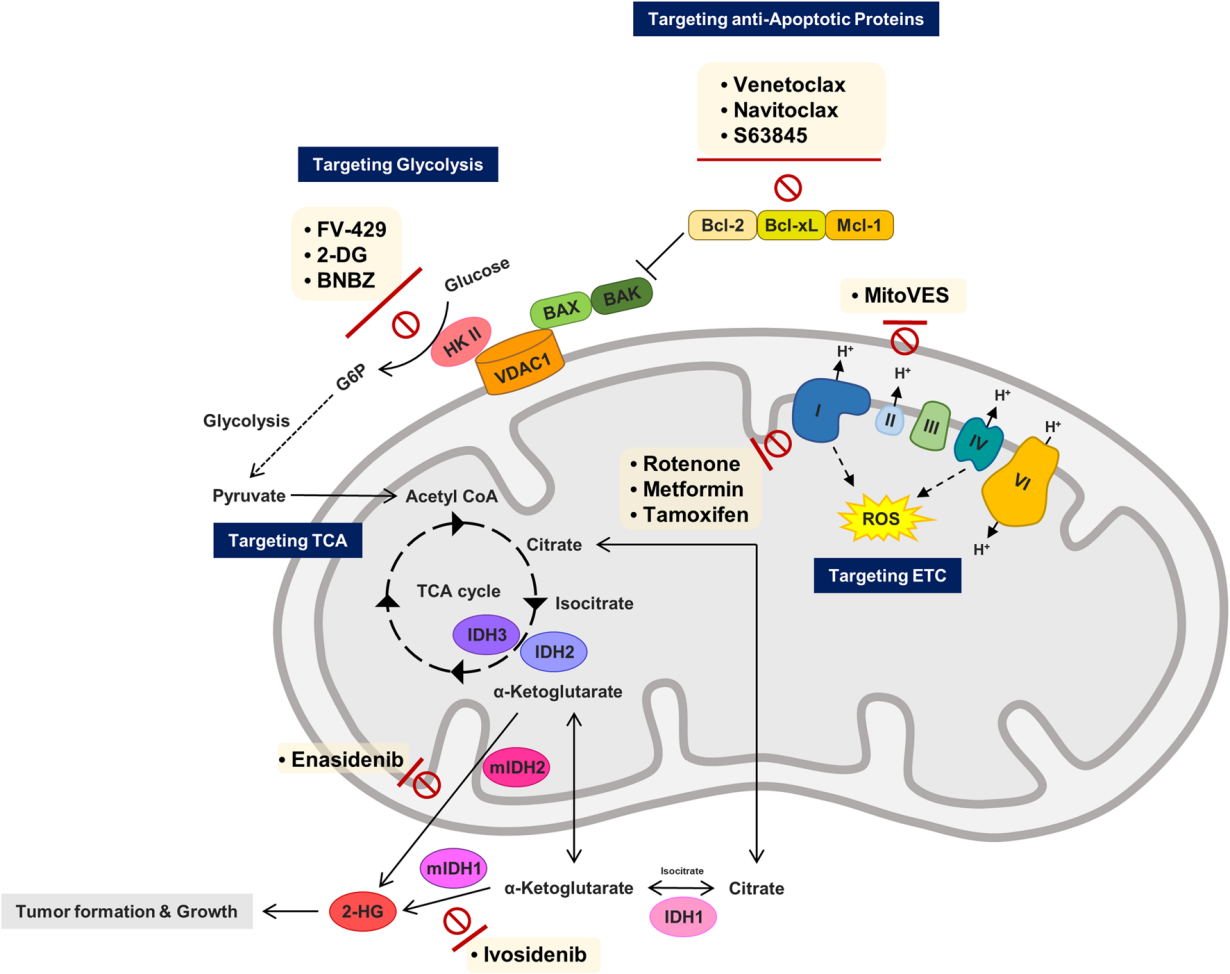
Cancer drugs that inhibit protein function involved in mitochondrial metabolism and anti-apoptosis

Mitochondrial dysfunction is a major cause of tumorigenesis and tumor progression in many cells. Many anticancer drugs that target dysfunctional mitochondrial metabolism and apoptosis pathways have been developed (Fig. 1). However, intrinsic genetic mutations and mutations in the target protein that are induced by continuous administration of anti-cancer drugs can cause induction of resistance. Administration of multi-drug therapy is recommended as a method for overcoming resistance to anti-cancer drugs. Resistance was overcome with use of a combination of mitochondrial-targeting drugs (Cheng et al. 2019). However, there are both advantages and disadvantages associated with overcoming drug-resistance. The primary risk is side effects. A better effect was not achieved by combination of valproic acid with all-trans retinoic acid, and valproic acid-related hematologic toxicity and higher mortality were observed with co-administration of idarubicin in patients with AML (Tassara et al. 2014). In addition, patients may show separate side effects for each drug at the same time, and determining which drug caused the side effect can be difficult (Mokhtari et al. 2017). Thus, the significance of targeted therapy to minimize toxicity may be undermined by the side effects of multi-drug therapy. In addition, increased drug costs due to increased usage of drugs and improper multi-drug use can impose a cost burden.

Positive combination therapy can have a synergistic effect on efficacy (Duarte and Vale 2022), however, use of sequential monotherapy may enable greater dose intensity as well use of a treatment approach that enables attainment of the maximum time and benefit from each agent (Dear et al. 2013).

Therefore, selection of an optimal mitochondria-target in order to minimize drug toxicity, identification of patients who show resistance to the drug to be administered, and design of an alternative strategy for treatment in patients who show resistance are important. Combination drug therapy or another single drug capable of evading resistance might be an alternative strategy. Finally, as various anti-cancer drug therapies have been developed, cases of resistance to the drug have also been reported; thus, conduct of many clinical studies is still required in various cases in order to achieve a successful treatment outcome for cancer patients.
